# Preliminary Findings of Elevated Inflammatory Plasma Cytokines in Children with Autism Who Have Co-Morbid Gastrointestinal Symptoms

**DOI:** 10.3390/biomedicines11020436

**Published:** 2023-02-02

**Authors:** Paul Ashwood

**Affiliations:** Department of Medical Microbiology and Immunology, School of Medicine, MIND Institute, University of California Davis, Davis, CA 95616, USA; pashwood@ucdavis.edu

**Keywords:** autism, ASD, immune, inflammation, gastrointestinal, comorbidities, cytokines, regulation, schizophrenia, tolerance, innate immunity, adaptive immunity, mucosal immunity

## Abstract

Autism spectrum disorder (AU) is present in approximately 2% of the population and is often associated with co-morbidities that can impact quality of life. One of the most common co-morbidities in autism is the presence of gastrointestinal (GI) symptoms consisting of irregular bowel habits such as constipation, diarrhea, or alternating bowel habit. Evidence of immune infiltration and immune activation has been shown in the ileum and colon of children with AU with GI symptoms. Moreover, immune dysfunction is a contributing factor in many GI diseases, and we hypothesize that it would be more apparent in children with AU that exhibit GI symptoms than those who do not present with GI symptoms. The aim of this preliminary study was to determine whether there are altered cytokine levels in plasma in children with AU with GI symptoms compared with children with AU without GI symptoms, typically developing (TD) children with GI symptoms and TD children without GI symptoms, from the same population-based cohort. Plasma cytokine levels were assessed by multiplex assays. No differences in plasma cytokines were observed in TD controls with or without GI symptoms; however, many innate (IL-1α, TNFα, GM-CSF, IFNα) and adaptive cytokines (IL-4, IL-13, IL-12p70) were increased in AU children with GI symptoms compared with children with AU with no GI symptoms. The mucosal relevant cytokine IL-15 was increased in AU with GI symptoms compared with all groups. In contrast, the regulatory cytokine IL-10, was reduced in AU with GI symptoms and may suggest an imbalance in pro-inflammatory/regulatory signals. These data suggest that children with AU and GI symptoms have an imbalance in their immune response that is evident in their circulating plasma cytokine levels. A finding that could point to potential therapeutic and/or monitoring strategies for GI issues in AU.

## 1. Introduction

Neurodevelopmental disorders, such as autism spectrum disorder (AU), are rapidly increasing in prevalence across the world. Their etiology is largely unknown but, in most cases, likely due to a combination of genetic and environmental factors. The diagnosis of AU is currently confined to behavioral traits including repetitive and stereotyped behaviors and by impairments in communication and social interaction. However, many individuals with AU also suffer from one or more medical comorbidities, including gastrointestinal (GI) dysfunction [1,2,3,4,5,6,7] GI symptoms in autism have been reported for 80 years [8], with irregular bowel symptoms of constipation and diarrhea, being the most common [9,10,11]. In our large cohort study of 1000 participants, children with AU were 6–8 times more likely to suffer from GI symptoms compared with age-matched typically developing children; in addition, GI symptoms were associated with poorer behavioral assessment scores [12]. Despite the frequent reports of GI dysfunction in AU, there is a lack of referral to specialized clinics or appropriate treatment of GI symptoms. The absence of studies into biological signatures in AU co-morbidities has also hindered strategies that could help to alleviate GI issues.

Mucosal immune cells comprise approximately 70% of the immune cells within the body, and dysfunction in these cells may have adverse consequences for GI function. Altered mucosal immunity can affect the host epithelial barrier function, the diversity of commensal bacteria in the gut and the enteric nervous system [3]. Many reports have described immune abnormalities in AU including changes in immune genetics, skewed cytokine production, altered T cell function and enhanced innate immune responses [13,14,15,16]. Moreover, immune activation has been reported in nearly two-thirds of children with AU and associated with more severe behaviors [13]. Endoscopic analyses of AU children with GI symptoms have revealed the presence of a subtle, diffuse inflammation of the intestinal tract (reviewed in [1,4]). However, the precise nature of this inflammation has been debated and is currently not clear. Histology, immunohistochemistry and flow cytometry evidence has consistently shown pan-enteric infiltration of immune cells such as lymphocytes, monocytes, natural killer (NK) cells and eosinophils into the walls of the GI tract in children with AU, compared with typically developing (TD) children with GI symptoms [9,17,18,19,20,21]. These reports have shown that in children with AU and GI symptoms, there are increased immune cell infiltrates in the colon, ileum, duodenum and stomach. Furthermore, the infiltrating lymphocytes exhibit a marked pro-inflammatory phenotype—with increased CD3^+^IL-6^+^ cells, CD3^+^TNFα^+^ cells and reduced regulatory CD3^+^IL-10^+^ cells in children with AU and GI symptoms compared with controls [17,18]. Comparing intracellular cytokine production pre- and post-immune challenge in peripheral T cells revealed a similar profile with increased production of pro-inflammatory cytokines but decreased regulation in children with AU who had GI symptoms compared with controls [22]. The stimulated cytokine responses seen in children with AU and GI symptoms were different from children with AU without GI symptoms as well as TD control children and suggest that cytokine production following immune challenge may be unique in children with AU and GI symptoms. The immune profiles in children with AU and GI symptoms were also different from children with established inflammatory bowel disease (IBD), such as Crohn’s disease, celiac disease and ulcerative colitis [9,17,18,19,21]. Similar findings of increased pro-inflammatory cytokine production after stimulation, IL-1β, IL-6 and TNFα, have been shown in children that have AU and food sensitivity compared with controls [23,24].

The search for biological signatures or biomarkers in autism has so far been an understudied area. Arguably the biggest utility of biological signatures in the context of AU will not be in determining core symptoms but of associated co-morbidities. These co-morbidities often have profound effects on severity of symptoms or associated behaviors such as sleep, irritability, aggression, or anxiety [3]. Some plasma cytokines or immune mediators have been investigated for their potential as biomarkers, mostly in the context of AU severity [14,25,26,27]. Plasma markers that could help identify or track the trajectory of GI symptoms in AU are lacking. In the current study we seek to characterize the plasma cytokine profiles in children with AU with or without GI symptoms from a random sampling of a population-based case–control study.

## 2. Materials and Methods

This study included 79 participants who were enrolled the CHARGE (Childhood Autism Risk from Genetics and Environment) Study, an ongoing population-based case–control study [28]. Briefly, CHARGE Study participants were selected from 2 strata: autism disorders (AU), and the general population typically developing (TD) controls. Eligible children were between the ages of 24 and 60 months, born in California, living with at least one biological parent who spoke English or Spanish, and residing in the catchment areas of a specified list of regional centres in California. Children with AU were identified through regional centres, providers/clinics, self-referrals, and general public outreach. TD children were identified from state birth files, and a stratified random sample was generated by frequency-matching to a projected distribution of AU cases on age, sex, and catchment area. Children with major motor and sensory impairments (e.g., blindness and deafness) that would preclude a valid developmental assessment were excluded. The CHARGE Study protocol was approved by institutional review boards of the University of California in Davis and the State of California Committee for the Protection of Human Subjects. Written informed consent was obtained before participation.

For this study, 40 cases with AU and 40 TD controls were randomly selected from a pool of CHARGE Study participants enrolled between April 2003 and April 2008, provided a blood sample, completed a GI History questionnaire, and had a confirmed AU or TD diagnosis. Cases and controls were matched on age at blood draw (at 3-month intervals) and whether or not they had frequent GI symptoms of irregular bowel movements (defined as frequent diarrhoea or constipation in the last 3 months). The initial goal of the study was to have 20 cases and 20 controls with frequent GI symptoms as well as 20 cases and 20 controls without frequent GI symptoms. Because at the time of the study only 9 controls in the entire CHARGE Study had frequent GI symptoms, cases and controls were only matched on age at blood draw.

The final sample of 80 children comprised 20 cases (mean age 41.7 ± 9.6 months; 16 males) and 9 controls (mean age 41.6 ± 9.1 months; 7 males) with frequent GI symptoms and 20 cases (mean age 41.9 ± 9.3 months; 19 males) and 31 controls (mean age 42.2 ± 9.3 months; 26 males) without frequent GI symptoms. As this was a random sampling, we did not exclude based on medication use. Few children were taking medications at the time of blood draw: these included antimicrobials such as acyclovir, ketoconazole (2 cases with GI symptoms, and 2 cases and 2 controls without GI symptoms), steroids such as asthma inhalers, nasonex (2 cases and 1 control with GI symptoms and 1 case and 1 control without GI symptoms), and GI-related medications such as miralax, nexium, milk of magnesia (2 cases and 3 controls with GI symptoms). None of the participants were taking anti-psychotic medications. We found no medication effects on the data when analyzed together (treated vs. nontreated, within groups), or for individual medications.

Autism diagnosis was confirmed using gold standard assessments: Autism Diagnostic Interview-Revised (ADI-R; [29]) and Autism Diagnostic Observation Schedule (ADOS; [30]). Controls were screened for AU using the Social Communication Questionnaire (SCQ; [31]) and none scored above the cut-off (SCQ ≥ 15). Mullen Scales of Early Learning (MSEL; [32]) and Vineland Adaptive Behavior Scales (VABS; [33]) were administered to cases and controls to determine cognitive and adaptive development, respectively. Typical development in controls was defined as having composite scores of ≥70 on both assessments and no previous diagnosis of developmental delay. All clinicians at the UC Davis MIND (Medical Investigation of Neurodevelopmental Disorders) Institute had attained research reliability on the developmental assessments they administered (ADI-R, ADOS, MSEL, and VABS). Bilingual study staff were available to administer informed consent and all instruments/questionnaires in Spanish.

### 2.1. Blood Collection and Cytokine Analysis

Peripheral blood was collected from each subject in acid-citrate dextrose Vacutainers (BD Biosciences; San Jose, CA, USA). Blood was centrifuged at 2100 rpm for 10 min, plasma was then harvested and stored at −80 °C prior to analyses of cytokines. Cytokine concentrations in the plasma of participants were determined by a multiplexing bead immunoassays assay (Millipore, Billerica, MA, USA). Samples and reference controls were run according to manufacturer’s protocol. Briefly, 25 µL of plasma sample was incubated with antibody-coupled fluorescent beads, then washed and incubated with biotinylated detection antibodies followed by streptavidin–phycoerythrin. The beads were then analyzed using flow-based Luminex™ 100 suspension array system (Bio-Plex 200; Bio-Rad Laboratories, Inc., Hercules, CA, USA). Standard curves were generated by Bio-plex Manager software to determine unknown sample concentration. All samples and reference cytokines were prepared according to the manufacturer’s recommendation and cytokine and chemokine levels were assessed by Luminex™ multiplex analysis. Values of samples are expressed as pg/mL. The cytokines/chemokines analyzed were interleukin (IL)-1α, IL-1β, IL-2, IL-3, IL-4, IL-5, IL-6, IL-7, IL-8, IL-10, IL-12 (p40), IL-12 (p70), IL-13, IL-15, IL-17, granulocyte macrophage colony stimulating factor (GM-CSF), granulocyte colony stimulating factor (G-CSF), interferon gamma (IFNγ), IFN-α2, tumor necrosis factor alpha (TNFα), TNFβ, eotaxin, monocyte chemotactic protein (MCP)-1, macrophage inflammatory protein (MIP)-1α, MIP-1β, and 10 kDa interferon-gamma-induced protein (IP-10), the minimum detection limits for these cytokines/chemokines were 2.4, 0.3, 1.2, 1.3, 2.2, 0.2, 1.1, 2.4, 0.9, 0.3, 34.9, 0.8, 1.0, 0.8, 0.4, 5.0, 0.9, 0.5, 0.4, 1.5, 0.9, 5., 1.8, 5, 10,7, and 3.6 pg/mL, respectively. Concentrations obtained below the limit of detection (LOD) of the method were calculated as half the limit of detection (LOD/2) for statistical comparisons. Values obtained from the reading of samples that exceeded the upper limit of the sensitivity method were further diluted and cytokine concentrations calculated accordingly. Intra and inter-plate/assay variations of cytokine levels, using representative samples run on all plates, were less than 5%. Plasma aliquots had not undergone any previous freeze/thaw cycle. The cytokine analyst was blinded to the case or control status of each sample.

### 2.2. Statistical Analyses

Cytokine and chemokine concentrations were natural-log-transformed for statistical analysis. Covariates of interest as possible confounders included the child’s age at blood draw. Immune responses can change with age; thus, it is important to adjust for age as a confounder. Analyses of covariance (ANCOVA), adjusted for age at blood draw, were performed to compare analyte concentrations in cases and controls with and without GI symptoms. Adjusted means and standard errors were presented as exponentiated values in pg/mL units. *p*-values were corrected for multiple comparisons using the Tukey–Kramer method and considered statistically significant if *p* < 0.05 after the corrections were applied. All analyses were performed using SAS version 9.2 (SAS Institute Inc., Cary, NC, USA).

## 3. Results

We found no differences in any cytokines between TD controls who had GI symptoms and those TD controls that did not have GI symptoms, after adjusting for child’s age at blood draw (Table 1). Among AU cases, compared to those without GI symptoms those with GI symptoms had significantly higher innate immune cytokines, including; IFNα-2 levels (adjusted mean 86.574 (standard error 1.234) vs. 38.092 (1.241) pg/mL, *p* = 0.04); IL-1α levels (23.999 (1.496) vs. 5.028 (1.513) pg/mL, *p* = 0.04); TNFβ (21.672 (1.349) vs. 5.094 (1.358) pg/mL, *p* = 0.006); and, IL-15 (3.561 (1.405) vs. 0.690 (1.418) pg/mL, *p* = 0.006). For cytokines mostly associated with adaptive lymphocyte responses, including; IL-2 (2.052 (1.391) vs. 0.525 (1.404) pg/mL, *p* = 0.03); IL-12p70 (5.989 (1.332) vs. 1.954 (1.342) pg/mL, *p* = 0.04); IL-4 (3.456 (1.432) vs. 0.669 (1.445) pg/mL, *p* = 0.01); and, IL-13 (1.747 (1.568) vs. 15.502 [1.551] pg/mL, *p* = 0.005), these were significantly elevated in AU cases with GI symptoms compared to those AU cases without GI symptoms. Interestingly, the regulatory cytokine IL-10 was decreased in AU cases with GI symptoms compared with those without GI symptoms (1.504 (1.516) vs. 9.365 (1.499) pg/mL, *p* = 0.01).

Mean GM-CSF concentrations were significantly higher in the AU with GI symptoms group compared with TD controls no GI symptoms after adjusting for child’s age at blood draw (16.248 (1.298) vs. 4.568 (1.298) pg/mL, *p* = 0.04; Table 1). Moreover, levels of other innate cytokines were increased for IL-1α (23.999 (1.496) vs. 9.757 (1.383) pg/mL; *p* = 0.011); IFNα-2 (86.574 (1.234) vs. 50.3(1.184) pg/mL; *p* = 0.026); TNFβ (21.672 (1.1.349) vs. 8.248 (1.271) pg/mL; *p* = 0.006) and IL-15 (3.561 (1.405) vs. 0.905 (1.314) pg/mL; *p* = 0.01) in AU with GI symptoms compared with TD controls without GI symptoms. AU children with GI symptoms had significantly higher IL-4 levels than TD controls with GI symptoms (0.542 [1.707], *p* = 0.03). Levels of IL-13 were also significantly higher in AU children with GI symptoms compared to levels in TD controls with GI symptoms (1.113 (1.923), *p* = 0.03). IL-15 levels were also significantly higher in AU children with GI symptoms compared with TD children with GI symptoms (0.499 (1.660), *p* = 0.01).

The concentrations of several other cytokines differed across cases and controls with and without GI symptoms but did not reach statistical significance at α = 0.05. Levels of IL-5 were higher in AU children with GI symptoms compared with AU children without GI symptoms (0.433 (1.504) vs. 0.106 (1.520) pg/mL, *p* = 0.08); moreover, IL-5 levels were higher in AU children with GI symptoms compared to TD children without GI symptoms (0.071 (1.839) pg/mL, *p* = 0.07). AU children with GI symptoms also had higher IL-6 concentrations than AU children without GI symptoms (3.487 (1.492) vs. 0.886 (1.507) pg/mL, *p* = 0.09), and AU children with GI symptoms had higher IL-6 concentrations than TD children without GI symptoms (0.583 (1.815) pg/mL, *p =* 0.07). Similarly, IL-7 levels were higher in AU children with GI symptoms compared with TD children without GI symptoms (7.637 (1.381) vs. 1.848 (1.619), *p* = 0.08).

## 4. Discussion

The immune system plays an essential role in protecting the host from infections and is continuously challenged by both external and internal stimuli. Inflammation is an important defense and survival response, triggered by innate and adaptive immune mechanisms. However, persistent inflammation or dysregulated immune response could lead to impairment of physiological process in both immune and non-immune systems. Moreover, inflammation leads to increased production of reactive oxygen species that can cause oxidative stress and tissue damage. In AU, we and others have observed alterations of immune-related genes, inflammatory markers, oxidative stress, immune cell activation and response to pathogens [3,5,10,34]. In this preliminary study, we found that children with AU and GI symptoms have elevated levels of innate cytokines IFNα, IL-1a, IL-15 and TNFβ, and adaptive cytokines IL-2, IL-4, IL-12 (p70), and IL-13, but decreased regulatory cytokine IL-10 compared with children with AU without GI symptoms. In TD controls we found no differences based on GI symptoms. AU children with GI symptoms had significantly higher IL-4 and IL-13 levels than TD controls with GI symptoms. Moreover, several innate cytokines were increased in children with AU with GI symptoms compared with TD controls without GI symptoms including, GM-CSF, IL-1α, IFNα-2 and TNFβ. Finally, the mucosal related cytokine IL-15 was also increased in AU with GI symptoms compared to both TD controls with and those without GI symptoms. These data suggest that there may be different patterns of plasma cytokines in children with AU dependent on the presence of co-morbidities such as GI symptoms.

The premise/utility of biological markers or signatures in AU is simple; to aid in diagnoses, to help monitor treatments/interventions, and to point to pathological pathways involved in causation(s). However, the implementation of biological markers into a research or clinical settings in AU is far from simple and has so far been largely understudied. For instance, in this study differences in plasma biomarkers were only apparent in the AU group with GI symptoms and not those without GI symptoms, after adjusting for age at child blood draw and statistical correction for multiple comparison. This may reflect a true difference in immune activation in the AU group with GI or that the AU group without GI is more heterogeneous. Based on the tools available in the CHARGE study (i.e., assessments using VABS, MSEL, ADOS and ADI-R;), we were unable to reveal further co-morbidities in AU such as anxiety, attention deficit hyperactivity disorders, or enlarged brain growth that may also have an immune basis in the participants in the study. These co-morbidities could be present in either AU group, however, based on the current assessments there was no substantial differences in scores between the two AU groups. Further studies with larger sample sizes are warranted to investigate whether other co-morbidities could also be identified using plasma cytokines. As well as differences within the AU groups, there were also differences between AU with GI and TD controls, with and without GI issues in plasma cytokine levels; this added further potential for plasma cytokines as biological signatures for the GI co-morbidities.

The increased production of IL-15 in AU with GI compared with AU without GI and both TD (with and without GI symptoms) has several implications for mucosal immune health. IL-15 is produced by epithelial cells in the gut and innate immune cells such as macrophages, and dendritic cells. IL-15 promotes T cell proliferation and cytokine production, influences the expression of the mucosal adhesion integrin—αEβ7—on intraepithelial T cells, and it can also induce proliferation of intestinal epithelial cells [35]. In the GI tract, IL-15 is overexpressed in the gut mucosa of patients with celiac disease and is thought to contribute to epithelial damage [36]. The health of the GI tract is highly dependent on an intact gut barrier function in part regulated by tight junctions located in-between the enterocytes. Using the lactulose:mannitol test, decreased intestinal permeability has been shown in autism [37,38]. Moreover, a previous study demonstrates that 75% of intestinal samples isolated from individuals with autism has reduced expression of barrier-forming tight junction components, and 66% had increased pore-forming claudins when compared to controls [39]. We have also shown altered levels in the genes controlling zonulin levels, a molecule that regulates intestinal permeability, in children with AU with GI symptoms but not AU without GI symptoms or controls [10]. IL-15 is also involved in the activation of NK cell a finding previously seen in autism [5]. Furthermore, IL-15 can block regulatory T cell generation by inducing IL-12p70 production in dendritic cells [40]. Decreased regulatory T cell formation have been observed in autism [22,41]. In the current study we also saw increased IL-12p70 in AU with GI symptoms.

Differences in other innate cytokines were also noted in AU with GI symptoms. The innate immune system acts as a first line of defense and is triggered through pattern recognition receptors such as Toll-like receptors (TLR). In the GI tract these interactions are important as there is potential to respond to commensal bacteria, food-borne pathogens, or bacterial by-products. Major cells of the innate immune system include macrophages, dendritic cells, NK cells and neutrophils. Perivascular macrophages and microglia (a specialized type of macrophage) are resident immune cells in the brain and provide protection against damage or infections. Alterations in innate response and microglia function are linked to several neurodevelopmental disorders, including autism [5]. Brain inflammation with activated microglia and astrocytes that affects neuronal connectivity with loss of synaptic connection and neuronal cell death has been described in ASD [42]. Studies also show elevated levels of pro-inflammatory cytokines such as IL-1β, IFN, TNF and IL-8 both systemically and in the brain [5]. Meanwhile anti-inflammatory cytokines, such as transforming growth factor beta1 (TGFβ1), IL-35 are decreased in AU [10,25,27,42]. Interestingly, multiple studies reported strong associations between the severity of AU-related behaviors and cytokine levels, [3,13,43]. Of note, innate cytokine IL-1 can affect the hypothalamic-pituitary-adrenal (HPA) axis, and IL-6 has important roles in early neurodevelopment and neuro-immune communication [5].

As well as a change in the balance of innate cytokines and IL-12, we also saw increases in cytokines associated with atopy in AU with GI symptoms. IL-4, and IL-13 are associated with humoral responses, and are drivers of inflammation in atopy and food allergies [40,46]. Autism has previously been associated with increased food allergies and asthma [44,45,46,47,48]. Increased IL-4 has also been found in neonatal blood spots from children who later developed severe AU [49]. Further studies to investigate biomarkers and food allergies in AU are warranted.

Furthermore, another difference between the AU groups was the reduced plasma levels of IL-10 and may suggests an imbalance in immune regulation. We, and others, have observed decreased plasma levels of other regulatory cytokines such as active TGFβ1 and IL-35 levels in adults and children with AU [25,27,50]. Several reports have also shown decreased IL-10 levels in T cells from children with autism [17,18,22,51]. Maintaining immune homeostasis is a balance between providing regulation/tolerance to self-proteins and important beneficial commensal microbes, and responding to pathogenic microbes. Previous studies investigating stimulated responses in immune cells in AU children, found that those who have marked behavioral fluctuations and GI symptoms, have decreased IL-10 to certain immune stimuli [23,24]. While increased inflammatory mediators help mount effective defense against pathogens, disruption in regulation can lead to excessive inflammation and has been implicated in many autoimmune disorders including those that affect the GI such as Crohn’s disease and ulcerative colitis [52,53,54,55].

Recent studies indicate that host immune responses can influence the gut microbiome, as well as the nervous system and may be involved in the pathology of AU [4]. For instance, the innate immune response can shape the microbiome composition through production of antimicrobial molecules including α-defensins and β-defensins, whereas adaptive responses can lead to IgA, and T cells that are specific for commensal bacteria [56,57,58]. Furthermore, the production of reactive oxygen species due to inflammatory responses can lead to overgrowth of specific bacterial species, altering the microbiome composition. Altered microbiota composition is commonly found in AU; however, the causes of this are unknown and may relate to food sensitivities or dietary preferences [59]. Moreover, it was recently shown that GI symptoms in AU are not associated with microbiome changes in AU [60], whereas we show here and previously, that immune activation and plasma cytokines are associated with GI symptoms in AU [10,22,25]. Further studies are needed to clarify the role of the immune–gut–brain axis, including the interplay between immune system, intestinal barrier function, microbiome, and the vagal nerve and peripheral nervous system.

As a preliminary study, there are several limitations. Our study is limited by small sample sizes that impacted any behavioral analyses within groups and restricted how we could stratify our study population. We did not find evidence for predictive markers of state and trait features with any cytokine using area under the curve (AUC) analyses (data not shown) a fact that most likely reflects the large number of variables that need correcting for and the small group sizes. As with other studies, the recruitment of TD children who experience GI issues was difficult due to their low frequency in the general population in this age group. We focused on symptoms of irregular bowel movements as these previously have been the most associated with GI symptoms in AU [1,3]. Due to limited numbers, we were unable to further break down groups to examine differences between specific GI symptoms clusters (e.g., constipation vs. diarrhea vs. IBS) but this warrants further study. As recruitment of males and females was consistent with AU diagnosis, we did not have sufficient statistical power to analyze sex differences due to the low number of females per group. Finally, our study included a narrow (young) age group, in follow up studies it would be warranted to compare plasma cytokines in older age groups to see how GI symptoms change and how they are associated with plasma cytokines across age. In addition, in this study we cannot determine the direction of elevated cytokine levels, i.e., is it generated in gut epithelium, lamina propria, mesenteric lymph nodes or liver. However, even with these limitations we feel that this study provides invaluable clues for plasma cytokines and GI symptoms in children with AU.

Due to the heterogeneity of AU and the varying types of immune dysfunction reported, we sought to investigate differences in plasma cytokines within a subgroup of children based on the comorbidity of GI symptoms. The main aim of this study was to identify differences in inflammatory and regulatory plasma cytokines in AU with and without GI symptoms compared with TD children. Children with AU and GI symptoms displayed the largest number of differences with elevated inflammatory cytokines and decreased regulatory IL-10 compared with AU with no GI symptoms. Mucosal relevant IL-15 was increased in AU with GI symptoms compared with all groups. We previously reported altered immune responses in children with AU who experience GI symptoms. Peripheral blood mononuclear cells from children with AU and GI symptoms produced increased mucosa-related cytokines but decreased active TGFβ1 after stimulation in vitro [10], suggesting a net imbalance away from a regulated response. These data, along with the current study, illustrate the need to find common subgroups within AU, that may help define more targeted treatments to benefit individuals across the spectrum [1]. The field of AU requires further investigation to elucidate the complex pathogenesis of this wide spectrum of conditions and co-morbidities. This leads to the question of how we can ameliorate intestinal disorders in the context of AU. For example, can immune-modulation help restore intestinal homeostasis and which immune-related factors should be targeted? Our data suggest two possible areas, either decreasing inflammatory cytokines or increasing immune regulation. Future studies could focus on immune cells activation in the gut in AU to help unravel signaling pathways, and immune activation in children with AU with GI co-morbidities.

## Figures and Tables

**Table 1:** Comparison of mean log-transformed cytokine and chemokine concentrations (pg/mL) in AU and TD groups with and without GI symptoms *.

	AU no GI (n = 19)		AU with GI (n = 19)		TD no GI (n = 31)		TD with GI (n = 9)							
pg/mL	Adj. Mean	SE	Adj. Mean	SE	Adj. Mean	SE	Adj. Mean	SE	*p*-Value ^1^	*p*-Value ^2^	*p*-Value ^3^	*p*-Value ^4^	*p*-Value ^5^	*p*-Value ^6^
Eotaxin	72.096	1.138	84.775	1.134	76.784	1.207	67.830	1.106	0.8063	0.9377	0.9927	0.5155	0.9823	0.9715
G-CSF	100.786	1.220	146.936	1.214	139.212	1.335	151.866	1.169	0.5318	0.9935	0.7950	0.9992	0.3742	0.9987
GM-CSF	9.061	1.307	16.248	1.298	4.568	1.476	8.750	1.234	0.4058	0.4600	0.4724	0.2583	0.9996	**0.0407**
IFNα-2	38.092	1.241	86.574	1.234	29.137	1.369	50.300	1.184	**0.0395**	0.4222	0.8943	0.1935	0.7419	**0.0258**
IFNγ	1.931	1.441	6.228	1.428	1.865	1.701	3.435	1.331	0.1088	0.7422	0.9999	0.5646	0.6038	0.2427
IL-1α	5.028	1.513	23.999	1.496	2.425	1.824	9.757	1.383	**0.0412**	0.1832	0.7501	0.3105	0.5899	**0.0117**
IL-1β	2.869	1.279	5.618	1.270	2.375	1.429	4.011	1.212	0.2118	0.5702	0.9722	0.6920	0.7050	0.1956
IL-2	0.525	1.404	2.052	1.391	0.619	1.636	0.882	1.303	**0.0259**	0.9202	0.9925	0.2004	0.6225	0.1889
IL-3	6.259	1.115	6.117	1.112	6.931	1.171	6.534	1.089	0.9988	0.9878	0.9515	0.9629	0.9895	0.9142
IL-4	0.669	1.445	3.456	1.432	0.542	1.707	0.933	1.334	**0.0108**	0.8084	0.9882	**0.0287**	0.8922	**0.0264**
IL-5	0.106	1.520	0.433	1.504	0.071	1.839	0.156	1.388	0.0844	0.6589	0.9468	0.2198	0.8824	0.0724
IL-6	0.886	1.507	3.487	1.492	0.583	1.815	1.540	1.379	0.0877	0.4810	0.9380	0.3896	0.7135	0.0694
IL-7	2.633	1.394	7.637	1.381	1.848	1.619	3.593	1.297	0.1072	0.6189	0.93002	0.2730	0.8810	0.0773
IL-8	2.641	1.505	3.740	1.489	2.160	1.811	3.888	1.377	0.9286	0.8193	0.9923	0.9998	0.8783	0.8685
IL-10	1.944	1.829	3.823	1.385	1.504	1516	9.365	1.499	**0.0123**	0.7582	0.9852	0.3190	0.2977	0.1438
IL-12(p40)	24.386	1.318	36.489	1.309	19.826	1.495	26.977	1.241	0.7239	0.9062	0.9743	0.8188	0.9915	0.5906
IL-12(p70)	1.954	1.342	5.989	1.332	2.128	1.533	3.971	1.259	**0.0389**	0.5751	0.9984	0.6785	0.2384	0.1926
IL-13	1.747	1.568	15.502	1.551	1.113	1.923	3.174	1.423	**0.0047**	0.4978	0.9415	**0.0310**	0.7244	**0.0070**
IL-15	0.690	1.418	3.561	1.405	0.499	1.660	0.905	1.314	**0.0064**	0.7301	0.9524	**0.0126**	0.9278	**0.0100**
IL-17	0.965	1.402	2.683	1.391	1.124	1.636	2.286	1.303	0.1424	0.5846	0.9940	0.9814	0.1943	0.4611
IP-10	174.688	1.296	263.223	1.287	193.446	1.456	202.553	1.225	0.6701	0.9996	0.9961	0.8486	0.9700	0.9040
MCP-1	158.064	1.254	247.646	1.246	203.365	1.388	180.910	1.194	0.4889	0.9890	0.9210	0.6819	0.9658	0.9593
MIP-1α	10.095	1.314	18.634	1.305	6.794	1.487	11.752	1.239	0.3792	0.6185	0.8438	0.5330	0.9715	0.1582
MIP-1β	31.375	1.171	46.993	1.166	30.084	1.257	37.751	1.131	0.2664	0.8195	0.9987	0.6834	0.7941	0.3756
TNFα	6.328	1.213	10.816	1.208	7.294	1.324	8.348	1.163	0.2054	0.9743	0.9757	0.7103	0.6742	0.65239
TNFβ	5.094	1.358	21.672	1.349	3.532	1.560	8.248	1.271	**0.0062**	0.3436	0.9051	0.0644	0.6059	**0.0062**

* Adjusted for age at blood draw; SE = standard error; *p*-values corrected for multiple comparisons using the Tukey–Kramer method. ^1^ Comparison of AU cases with GI and without GI symptoms. ^2^ Comparison of TD controls with GI and without GI symptoms. ^3^ Comparison of AU cases without GI and TD controls without GI symptoms. ^4^ Comparison of AU cases with GI and TD controls with GI symptoms. ^5^ Comparison of AU cases without GI symptoms and TD controls with GI symptoms. ^6^ Comparison of AU cases with GI symptoms and TD controls without GI symptoms. Significant results highlighted in bold.

## Data Availability

Data available on request.
